# “To be, or not be… satisfied in NPOs”: a serial multiple mediation and clustering analysis of paid staff and volunteers' profiles

**DOI:** 10.3389/fpsyg.2023.1042722

**Published:** 2023-07-11

**Authors:** Rocío López-Cabrera, Francisco J. Medina, Martin Euwema, Alicia Arenas

**Affiliations:** ^1^Department of Social Psychology, University of Seville, Seville, Spain; ^2^Occupational and Organizational Psychology and Professional Learning, Katholieke Universiteit Leuven (KU Leuven), Leuven, Belgium

**Keywords:** non-profit organizations (NPOs), conflicts, job satisfaction, job performance, serial multiple mediation, cluster analysis

## Abstract

**Introduction:**

Non-profit organizations (NPOs) are a complex working context whose main characteristic resides in the dichotomy between paid staff and volunteers. Despite its benefits for goal achievement, this circumstance can be also a challenge, for both groups' interaction, for their comprehension of their own role and to HR management. The aim of this study was to explore factors that may contribute to promote job satisfaction among NPOs' members.

**Methods:**

Combining two different data analyses, serial multiple mediation analyses and cluster analyses, first we analyze whether there are differences between paid staff and volunteers in perceived intrapersonal conflict and performance and its effect on job satisfaction, and second, we analyze whether there is an additional profiles distribution that reflects more adequately the reality of NPOs, despite the formal roles that coexist in these organizations (paid staff and volunteers).

**Results:**

Results confirm that paid staff and volunteers differ on their perceived job satisfaction level, with volunteers being more satisfied. This relationship is serially mediated by role conflict, role ambiguity, and performance. Another characteristic of the NPOs is that the dichotomy between paid staff and volunteers does not capture well the reality of the labor relations between members of both groups and the organization. To explore this phenomenon, we perform a cluster analysis based on paid staff and volunteers' perceptions. Cluster analyses demonstrate the existence of three rather homogenous profiles.

**Discussion:**

Additionally, practical implications for HR management in NPOs and future research lines to understand this organizational context dynamics are also discussed.

## 1. Introduction

Nowadays, managing diversity at work is one of the main challenges and goals of organizations and international institutions such as the European Commission and the United Nations (European Commission, 2021). Diversity is usually analyzed from an individual perspective, considering differences between social groups' characteristics (Van Knippenberg and Schippers, 2007). However, it can also derive from coexisting role identities or groups of employees with different needs within organizations (Arenas et al., 2017).

A clear example of this source of diversity are non-profit organizations (NPOs). Non-profit organizations (NPOs) are characterized by their intricate organizational structure and their heterogeneity, mostly defined by the coexistence of two main groups of employees: paid staff and volunteers (Studer and von Schnurbein, 2013). This dichotomy is a distinguishable positive feature of NPOs, as volunteers are a valuable resource to reach organizational objectives and they can exert a positive influence on organizations and users (Haski-Leventhal et al., 2011). However, at the same time, it can also be a source of conflict due to tensions between both roles; paid staff and volunteers usually have different perspectives on processes and working situations that contribute to the complexity of their interactions (Studer and von Schnurbein, 2013; López-Cabrera et al., 2020).

To guarantee NPOs' functioning, paid staff and volunteers are “condemned to get along” with each other, as they must complement their activities. Paid staff is responsible for management and coordination, monitoring complex activities and supervising tasks related to their professional background, whereas volunteers collaborate on the different projects insomuch as they have availability to be involved and committed to the organization (Ariza-Montes et al., 2015, 2017). However, despite paid staff responsibilities on daily management, volunteers are considered a core value in NPOs. Volunteering is considered part of the foundations of these organizations together with working toward a mission, usually based on providing help and support to others (Wilson, 2000). Therefore, volunteers also have responsibilities in decision-making bodies in the organization and usually set the direction of organizational aims and values.

Overall, in real practice, boundaries between paid staff and volunteers' roles are not that easy to differentiate in NPOs, even for both parties involved, and eventually, role discrepancies are a common issue in this work context. It is not unusual that paid staff report the feeling of being replaced by volunteers and volunteers of being replacing paid staff, respectively (Mook et al., 2014). Indeed, volunteer involvement in NPOs can also contribute to impairing paid staff motivation to accomplish their duties and their person–organization (P-O) fit (Jin et al., 2018), as well as their professional identity and their role characteristics and activities (Follman et al., 2016).

To understand this situation, it is also necessary to consider the organizational change that NPOs are facing because of financial struggles. In NPOs, social labor is a relevant source of motivation, satisfaction, and even organizational identity for their members, especially for volunteers (Warburton et al., 2018). Indeed, in these organizations, even efficiency has been traditionally relegated in favor of values such as participation or integration (Kreutzer and Jäger, 2011; Smith, 2018). However, in the last years, to respond to the current social changes and survive in a competitive environment, this paradigm is changing. NPOs are suffering a professionalization process to guarantee acquiring enough resources for their survival (Maier et al., 2016; Müller-Stewens et al., 2019; Currie et al., 2022). Consequently, paid staff increased their influence. A clear example of these changes is governments setting standards and procedures to identify and finance the most effective NPOs (Salamon, 2015) when they need to rely on third parties to manage humanitarian crises and provide basic social services to the community (Henriksen et al., 2012; Clausen, 2021). An example of this assistance has clearly taken place during the COVID-19 pandemic, when NPOs had an important role in supporting those in need not only due to health-related reasons but also social and economic consequences of its associated measures (Santos and Laureano, 2021).

These organizational changes imply the acquisition of a new “managerial” perspective, which is usually non-existent or ineffective in NPOs, and entails additional job demands particularly from paid staff and volunteers (Berzin and Camarena, 2018; McAllum, 2018; Petrella et al., 2021). In this context, adapted HR and human capital policies are essential, not only to promote successful programs but also to guarantee an optimal paid staff and volunteers' job satisfaction as well as an adequate performance (Ridder et al., 2012; AbouAssi et al., 2022). In previous research, López-Cabrera et al. (2020) stated that the complexity of NPOs organizational structure and the variety of tasks that they accomplish—sometimes considered incompatible activities—as well as their different aspirations and perspectives—even on the same aspects of their work—lead to disputes, interpersonal conflicts, and subsequent negative emotional consequences (López-Cabrera et al., 2020). Particularly, both paid staff and volunteers find it difficult to understand what is expected from their own positions, which leads to intrapersonal conflicts, such as role conflict and role ambiguity. Indeed, both groups reported this circumstance when describing process conflict, disagreement about how a task should be accomplished, including issues such as who should do what and how much responsibility each member of the group should take (Jehn, 1997; Jehn and Mannix, 2001).

However, due to new circumstances in the NPO's economical context, it is relevant to analyze the organizational processes that facilitate the reduction of rotation and promote staff and volunteers' organizational commitment and, overall, satisfaction. In this regard, it is essential to consider the differences between paid staff and volunteers and their perspectives. Particularly, a better understanding of what contributes to promote job satisfaction among these groups may determine how to manage their differences and needs. Otherwise, NPOs face the serious risk of losing members, particularly among volunteers who are not bonded by a contract (Bittschi et al., 2019).

To do so, in this study we first paid special attention to both roles—paid staff and volunteers, their perceived intrapersonal conflicts, and their effect on performance and job satisfaction. To provide HR managers with valuable information to understand their workforce (including volunteers), we examine the existent differences between both roles, regarding their perceptions of the organizational context and personal characteristics, inferring homogenous patterns or profiles that may help HRM to manage their organizations properly, being able to reach their goals.

All in all, this study is based on both literature review, as previously described, and the identification of needs of NPOs context in a previous study (López-Cabrera et al., 2020), both highlighting different perception on intrapersonal conflicts and their consequences as new challenges of Non-profit Organizations. These challenges are mainly provoked due to the intrinsic diversity of NPOs based on the dichotomy of roles, volunteers, and paid staff and the changes NPOs are facing in recent years to cope with context demands (Maier et al., 2016). This situation leads to professionalization; shifting the volunteering-based rationale as paid staff must assume administrative procedures (McAllum, 2018), as they require constant supervision and volunteers, usually has a very flexible work schedule combining their activity with remunerated job positions (Ariza-Montes et al., 2015, 2017). This change of paradigm can lead not only to interpersonal conflicts between both roles but to different perception regarding these conflicts and their consequences between paid staff and volunteers. Among these, consequences on interpersonal conflicts, job satisfaction, and performance outstand. The former due to its connections to well-being and intention to leave, and the latter because that prosocial work for helping others seems to have on both paid staff and volunteers encouraging job satisfaction and acting as a protection when paid staff and volunteers face intrapersonal conflict. The hypothesized relationships between these variables are stated in the forthcoming sections.

### 1.1. Research questions

The aim of this study is to explore factors that may contribute to promote job satisfaction among NPOs members. In this regard, we propose two main research questions.

First, we analyze whether there are differences between paid staff and volunteers in perceived intrapersonal conflict and performance and its effect on job satisfaction. Previous studies on this matter pointed out the differences between paid staff and volunteer's perception, particularly regarding interpersonal conflict and its negative consequences (López-Cabrera et al., 2020). These results remark pronounced differences on process conflict and specifically on conflicts related to roles (paid staff and volunteers), such as who is in charge or responsible for different tasks. Therefore, in this study we analyze whether being part of paid staff or volunteer's collective in NPO affects their perceived intrapersonal conflict (role conflict and role ambiguity), and consequently, each group's performance, and job satisfaction.

Second, and because of NPO internal characteristics, we explore (a) whether there is an additional profiles distribution that reflects more adequately the employment circumstances of paid staff and volunteers, despite their formal roles in these organizations, and (b) what are the effects of different profiles over conflict, performance, and job satisfaction. Managing this information, policies and human resources management practices can be adapted according to the real needs of organizational members, optimizing paid-staff and volunteer's management to increase their satisfaction and finally reduce NPO dropout.

### 1.2. How paid staff and volunteers perceive intrapersonal conflicts: consequences for performance and job satisfaction

Due to the changeable scenario that NPOs are currently facing (Maier et al., 2016; King, 2017), there is a certain ambiguity about responsibilities and tasks. Neither paid staff nor volunteers are usually sure about their duties and responsibilities in their working projects, the boundaries and relation between them. Therefore, to guarantee an appropriate organizational functioning it is relevant to understand how both paid staff and volunteers manage this uncertainty in their job and the potential conflicts this contains (King, 2017).

Previous research reported several problems in this matter. The recruitment of paid staff in some NPO due to professionalization processes may clash with their internal culture. At the same time, NPO goals require a high level of specialization guaranteed only by expert employees. For instance, paid staff believe that, to some extent, they are being replaced by volunteers; they consider that the essential activities in NPOs should be led by permanent staff and volunteering should be a supportive role (Mook et al., 2014). However, this perspective or change in roles distribution is not consistent with most NPO's culture, whose origins and development are usually based on volunteering.

However, volunteers usually lack specific training to deal with vulnerable users, so paid staff must monitor their activity, besides their multiple office tasks. Indeed, paid staff also think that they are who received the required formal social intervention training and that should be the main reason why they were hired; therefore, it should also be their main function as employees (López-Cabrera et al., 2020). Volunteers also feel they are being set aside by paid staff, as they are requested to support administrative duties and coordinating tasks. They want to be involved in social attendance; however, they consider that paid staff are also gaining relevance in this matter (McAllum, 2018). Overall, both paid staff and volunteers report discrepancies between what they expect to do and what they do in their positions.

Thus, considering NPOs' particularities, consequences on job satisfaction can vary depending on the role (paid staff or volunteer) as responsibilities and expectations are different for both groups. As Borzaga and Tortia (2006) pointed out, volunteers are protected by intrinsic aspects of their work in their organizations, and they feel rewarded just by being able to offer support to people in need. They are not contract bonded with the NPOS, so when they do not agree with the conditions of their collaboration, they might feel free to leave the organization (López-Cabrera et al., 2020). Therefore, to promote engagement among volunteers, satisfaction with their activities is crucial (Nagel et al., 2020). However, although job satisfaction can contribute to paid staff engagement, they are also bonded to the organization by a working contract that increases their obligations and limits their possibilities of leaving the organization without facing personal or even legal consequences. This is an extra burden for paid staff that can contribute to highlight differences in job satisfaction compared with volunteers. Therefore, we hypothesized that:

*H1: Paid staff reports less job satisfaction than volunteers*.

Considering the role of NPOs as a predictor of job satisfaction, it is important to understand the mechanism or processes involved in these differences between paid staff and volunteers. As explained by role theory (Kahn et al., 1964) when expected behaviors can be considered ambiguous or accountabilities are not clear, these circumstances would lead to role conflicts and role ambiguity among both groups, paid staff, and volunteers. Therefore, these two processes can contribute to explain the differences in job satisfaction between both groups.

Role conflict takes place when a person is expected to fulfill the duties of two contradictory positions; that is, there is a lack of compatibility between expectation and reality from a job or position (Rizzo et al., 1970). This situation usually comes, for instance, from incompatible demands requested by coworkers or supervisors, incompatible pressures due to membership in multiple groups, or conflict between personal values and activities related to the role (Tarrant and Sabo, 2010).

Concerning NPOs, this intrapersonal conflict takes place especially among paid staff, for example, when social workers or psychologists are expected to oversee administrative procedures and to get involved in social intervention (Tham, 2018; López-Cabrera et al., 2020). Volunteers, to a different degree, may also face this problem, particularly those who are involved in decision-making processes, considering that the actual shift to professionalization is clashing with the volunteering values of the organization (Beaton et al., 2021). Those collaborating on projects with paid staff may even feel they are taking away a job position as roles are too vaguely established (Hasenfeld, 2010; Overgaard, 2015). These circumstances may contribute to decrease job satisfaction.

Thus, we hypothesize that:

*H2: The relation between organizational role (paid staff or volunteer) and job satisfaction is mediated by role conflict*.

Besides fulfilling tasks that contribute to blur the limits between paid staff and volunteers' expected competencies (McAllum, 2018), NPOs members also must deal with new procedures implemented as part of organizational changes such as professionalization (Müller-Stewens et al., 2019). For instance, projects linked to public funding management entail the implementation of new economic justification procedures and additional administrative duties that are confusing for both paid staff members, who are trained in social intervention instead of in management, and volunteers, whose aim in the organization is usually to provide support to people in need (López-Cabrera et al., 2020). These circumstances can be conceptualized as role ambiguity, defined as the lack of clear information about tasks, methods, and consequences of role performance, and it is usually related to organizational complexity and organizational changes (Rizzo et al., 1970).

Consequently, paid staff and volunteers' experience of role conflict and role ambiguity generates uncertainty that has negative consequences not only for both groups, affecting their job satisfaction, but it can also be detrimental for goal achievement and performance. Indeed, as previously mentioned, studies confirm that role conflict is a significant (negative) predictor of job satisfaction (Carpenter et al., 2003, 2015; Belias et al., 2015) to the extent that it also can be linked to intention to leave (Mor-Barak, 2015). This is also the case for role ambiguity, which is also considered a source of dissatisfaction (Pousette, 2001). In this study, role conflict is considered as an antecedent of role ambiguity, as NPOs' paid staff and volunteers' dichotomy leads to inconsistencies between expectations and reality between both positions (López-Cabrera et al., 2020). Previous results reported that role conflict has a positive and significant effect on role ambiguity (Hartline and Ferrell, 1996). Indeed, conflicting expectations—role conflict—lead to uncertainty about execution and prioritization of those expectations—role ambiguity (Michaels et al., 1987). More recently, Pei-Lee et al. (2012) found out that role conflict is positively related to role ambiguity. Indeed, according to their results, role conflict fully mediated the effect of several total quality management practices on role ambiguity. Therefore, we hypothesized that the perception of role conflict and role ambiguity, together, may also influence paid staff and volunteers' job satisfaction (Koustelios et al., 2004), having a serial effect.

*H3: Paid staff and volunteers' differences on job satisfaction are serially mediated by role conflict and role ambiguity*.

Although managing role conflict and role ambiguity could be crucial to guarantee job satisfaction, performance in NPOs can impact paid staff and volunteers' job satisfaction, particularly considering that it usually implies social assistance (Treinta et al., 2020). Previous research stated that performance drives job satisfaction and vice versa (Hsieh, 2016). However, in NPOs performance is usually based on the perception of the success of social projects and activities that can help others in need (López-Cabrera et al., 2020); this positive expectation may have a buffering effect on the negative impact of role conflict and role ambiguity on job satisfaction. Therefore, in line with the results obtained by Christen et al. (2006), we consider that in NPOs, performance positively influences job satisfaction. Thus, in this organizational context, although there is lack of compatibility between expectation and their job reality that can reduce job satisfaction, being able to help others may buffer this negative effect.

Therefore, we hypothesize that:

*H4: Paid staff and volunteers' differences on job satisfaction are serially mediated by role conflict and role ambiguity and performance*.

**Figure 1:**
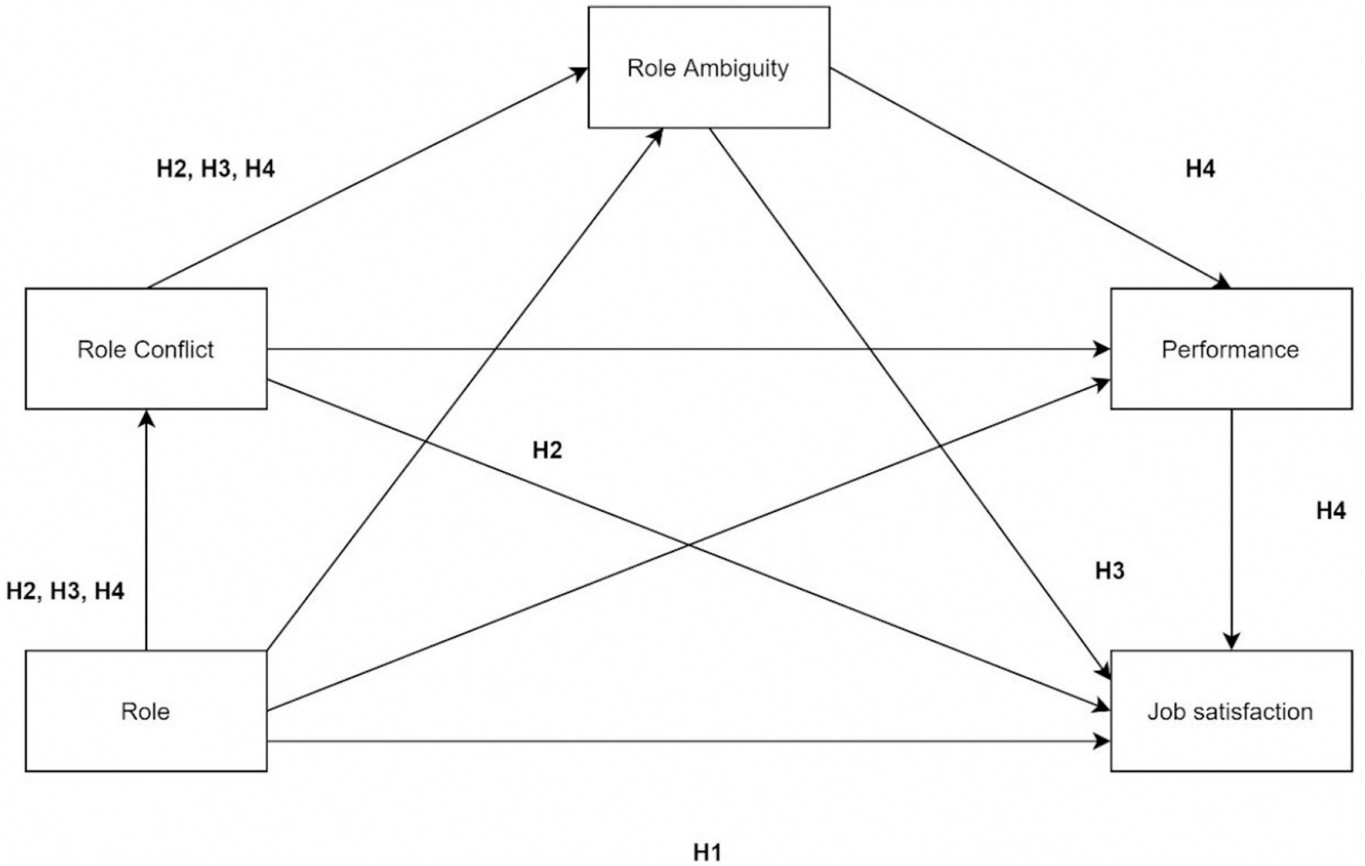
Theoretical model and hypotheses.

The theoretical model and hypotheses are presented in Figure 1.

### 1.3. Getting to know NPOs workforce: the importance of profile patterns

From an organizational intervention perspective, it is crucial that HRM knows the characteristics of their organizational members to promote their satisfaction at work and increase their performance (Bastida et al., 2018). However, NPOs are very heterogeneous, not only considering the existence of two different roles, but these groups are also diverse themselves (Netting et al., 2008). Paid staff encompasses different professional backgrounds such as psychologists and social workers, working on social projects, together with lawyers or economists occupying management-oriented positions. Volunteers, besides different educational backgrounds, may also have very diverse educational levels (primary education to doctorate), working situations (student, employed, unemployed, retired), and sociodemographic profiles, including age, previous experience dealing with social intervention, or seniority in the organization (McAllum, 2018).

Additionally, paid staff and volunteers cannot be considered stable categories, as a person can be temporarily hired by the NPO for a project and, at a certain time, continue his/her work as a volunteer in the same organization once his/her working contract ends. The opposite case is also feasible, and volunteers can be hired, at a certain moment, becoming part of paid staff. For instance, this usually happens with young volunteers that may have access to a job position once they have finished their studies. For this reason, to analyze the differences between these two categories, paid staff and volunteers, it is not possible to solely do it by contemplate whether the person has a contract at the time they are being interviewed.

Instead, to understand these differences between volunteers and paid staff we intend to analyze profiles in NPOs rather than isolated dimensions. Going beyond volunteers and paid staff categorization, it is possible to provide HR managers with valuable information based on their perceptions and interiorization of organizational procedures, to identify needs and problems in advance. As criteria variables to create these profiles, we considered perception of intrapersonal conflict (role conflict, role ambiguity, and role overload), conflict consequences (job satisfaction, job performance), and sociodemographic variables (age, seniority in the NPO, seniority in the project). Among intrapersonal conflicts, we also added role overload to profile analysis. Previous meta-analyses stated that role overload, unlike role conflict and role ambiguity, is non-significantly related to conflict consequences such as the level of performance (Örtqvist and Wincent, 2006; Eatough et al., 2011). However, this relation requires further exploring in research (Tang and Vandenberghe, 2021). Indeed, in profile analysis in NPOs, role overload can be relevant due to its connection with role ambiguity. The lack of clarity regarding paid staff and volunteers' responsibilities can contribute to induce both groups to accomplish more tasks than they are able to do in a particular time frame. This situation can impact job performance and job satisfaction, being even more relevant when those suffering from this overload have been working in the organization for a long period. These variables have been studied in previous research and proved to be relevant for HRM (e.g., Bastida et al., 2018; Bittschi et al., 2019; López-Cabrera et al., 2020).

## 2. Method

### 2.1. The participant NPO

In this study, we examined a large, representative, and worldwide NPO, particularly focusing on one of the main regional divisions, located in one of the largest regions in Spain. This regional division is composed of 20 local divisions, where 806 paid staff members and 8,442 volunteers work together on different social projects. We decided to focus on one large division of a worldwide NPO as it replies to the same functional and hierarchical structure in every division at both national and international levels. This NPO is worldwide present, so the results obtained in this study can be applied to all its divisions.

These two roles are intricately connected on their both functional and hierarchical structure. The main democratic decision-making bodies, at both regional and local levels, are known as “Committees”. These Committees are responsible for guarantee the observance of the general objectives, policy, strategy, and criteria established by the Institution's higher bodies. The top positions of these Committees are occupied by volunteers (named “Presidents”). Additionally, paid staff members are responsible of daily activities and strategic decisions concerning the ongoing social projects.

Regarding the different social projects, volunteers and paid staff are part of the same teams, although their activities are influenced by their roles: volunteers who present a very heterogeneous profile (in terms of age, working experience, professional background, or availability) collaborate part-time with a flexible schedule. However, a reduced number of paid staff members coordinate these projects, overseeing both administration and social intervention, and supporting and guiding volunteers on their activity. Concerning their professional profile, paid staff in this NPO's social projects are usually social workers and psychologists who have plenty of experience in this social context, particularly dealing with vulnerable groups and users in social risk.

This NPO replies to its hierarchical and functional structure on every division worldwide; therefore, these results can be applicable to all of them.

### 2.2. Participants

After receiving the invitation of the NPO HR and Volunteering Departments respectively, a total of 161 participants agreed to take part in the study. Data collection was conducted both online and using hard-copy questionnaires. Online questionnaires were distributed by email, using Qualtrics. Hardcopy questionnaires were collected after a mediation training session, organized by the NPO and the research team. Participation in this activity was optional and open to all the organization members.

After data processing, 113 questionnaires were eligible for further analyses, as it is described in the procedure section. Therefore, a total of 39 paid staff members (men =13; women = 26) and 74 volunteers (men = 26; women = 43) participated in this study. Regarding their educational background, paid staff members are university graduates, most of them on health and social sciences. Most volunteers were also university graduates; however, this group reports a more variety of educational background, including professional training, secondary and primary education, and occupations, such as civil servants, housekeepers, or retired professionals. Regarding participants' age, on the one hand, paid staff are between 24 and 55 years old (M = 38.64; SD = 7.37), and on the other hand, volunteers are between 18 and 78 years old (*M* = 51.26; SD= 16.11), which is representative of the diversity in this organization, particularly among volunteers.

### 2.3. Measures

#### 2.3.1. Intrapersonal conflicts: role conflict, role ambiguity, and role overload

Intrapersonal conflict was measured based on three different variables: role conflict, role ambiguity, and role overload. Role conflict (e.g., “Are you required to do contradictory activities at work?”) (α = 0.82) and role overload (e.g., “Does your work have clear objectives?”) (α = 0.84) were measured using the Spanish version of the Copenhagen Psychosocial Questionnaire (COPSOQ) by Moncada et al. (2005). Role overload (e.g., “The workload is so high that it does not allow me to do everything right”) (α = 0.89) was measured using a Spanish adaptation scale by González-Rom and Lloret (1998) included in the Michigan Organizational Assessment Questionnaire (MOAQ, Cammann et al., 1979). Each scale consists of three items rated on a 5-point Likert scale ranging from 1= “Never” to 5= “Always.”

#### 2.3.2. Job satisfaction

Job satisfaction was measured with the Spanish adaptation of the 5-item version of Hartline and Ferrell (1996) scale by Benitez et al. (2007). This scale assesses different aspects of satisfaction at work (e.g., “Your satisfaction with the compensation that you receive for your work at this organization”, “Overall, are you satisfied with your job in this organization”) (α = 0.82). Items were rated on a 5-point Likert scale ranging from 1= “Dissatisfied” to 5= “Very satisfied.”

#### 2.3.3. Performance

Performance regarding their social projects was measured using 4-item scale used by Hempel et al. (2009). These authors originally retrieved these items from Ancona and Caldwell (1992) criteria to evaluate team performance (e.g., “Efficiency, quality, technical innovation, and work excellence”) (α = 0.89). Items were rated on a 5-point Likert scale ranging from 1= “Low” to 5= “Very good”.

### 2.4. Procedure

#### 2.4.1. Data collection

Data collection was conducted using validated scales with high reliability as stated above. We adapted the different terms and organizational language to the one use of the organization. First, a content analysis was conducted by the HR department and volunteering coordination in order to check the possible difficulties in understanding the overall questionnaire or specific items and avoid possible bias.

The criteria to choose research participants were both convenience and acceptance. The participant NPO selected those projects where teams are composed of both paid staff and volunteers and informed team managers to promote their team's participation. During the first phase of the data collection, participants received directly from the research group an invitation by email requesting their collaboration in the study by answering an online questionnaire using the software Qualtrics. To guarantee anonymity, traceable personal information, was not registered when participants accessed and voluntarily completed the questionnaire such as emails or names. Following this procedure, 97 questionnaires were collected, 48 from paid staff and 57 from volunteers. Additionally, we decided on to second phase of the data collection, new participants were invited to participate during in-person training in conflict resolution techniques developed at the Regional Assembly of the NPO. These participants completed the same questionnaire, this time in hardcopy, instead of online. Following the same anonymity procedure, hardcopy questionnaires did not include traceable personal information. A total of 12 paid staff and 55 volunteers agreed to voluntarily take part in this second data collection. Participants were, in any case, free to participate or decline the invitation; that circumstance introduces some randomization in the process.

In both data collections, participants were informed about the procedure beforehand. They were requested to give their explicit consent before starting to answer the questionnaire, and confidentiality and anonymity were guaranteed during the whole process. Altogether, 169 questionnaires were collected both online and hardcopy. Nevertheless, 56 responses were deleted based on the following criteria: (a) questionnaires were registered when participants accessed online but they did not complete them; (b) participants only answered to a very limited number of items, not completing even a single scale; and (c) participants had no experience in the organization by the time they completed the questionnaire. Finally, a total of 113 responses were processed, 39 paid staff members (men =13; women = 26) and 74 volunteers (men = 26; women = 43).

#### 2.4.2. Data Analyses

Skewness and kurtosis index were used to identify the normality of the data. Results suggested that data deviation from normality was not severe as the value of skewness and kurtosis index were below ±2 and ± 7, respectively (Byrne, 2010; Hair et al., 2010; Kline, 2011). Descriptive and correlation analyses were conducted. Results are presented in Table 1. To test our Hypotheses, two different analyses were conducted: serial multiple mediation analysis and cluster analysis.

**Table 1:** Descriptive statistics and correlations.

	**Total NPO**						**Paid staff**						**Volunteers**					
	**M**	**SD**	**1**	**2**	**3**	**4**	**M**	**SD**	**1**	**2**	**3**	**4**	**M**	**SD**	**1**	**2**	**3**	**4**
1. Role conflict	2.14	0.93					2.87	0.84					1.75	0.73				
2. Role ambiguity	1.99	0.84	0.54^**^				2.5	0.84	0.57^**^				1.72	0.72	0.25^*^			
3. Role overload	2.46	1.25	0.74^**^	0.56^**^			3.65	1.06	0.65^**^	0.50^**^			1.83	0.8	0.53^**^	0.30^**^		
4. Performance	3.42	0.78	−0.50^**^	−0.48^**^	−0.46^**^		3.1	0.87	−0.56^**^	−0.50^**^	−0.31^**^		3.59	0.67	−0.31^**^	−0.33^**^	−0.42^**^	
5. Job satisfaction	4.06	0.65	−0.58^**^	−0.53^**^	−0.55^**^	0.52^**^	3.6	0.61	−0.40^*^	−0.57^**^	−0.33^*^	0.41^**^	4.3	0.52	−0.42^**^	−0.27^*^	−0.28^*^	0.46^**^

** Correlation is significant at the 0.01 level (two-tailed);

* Correlation is significant at the 0.05 level (two-tailed).

##### 2.4.2.1. Serial mediation model

Serial multiple mediation analyses were conducted using the PROCESS macro for SPSS (Model 6, three mediators) designed by Hayes (2018), to analyze the relationship among conflicts related to the role (role conflict and role ambiguity) and two possible consequences: job performance and job satisfaction. This model estimates the direct and indirect effects of the independent variable (role) on the dependent variable (job satisfaction), also considering several mediators (role conflict–role ambiguity–performance). In the model, each mediator is being a cause of the other mediator serially.

Serial multiple mediation analyses were conducted instead of other statistical methodology such as structural equation modeling (SEM) based on: (a) our sample size, which is not large enough to prevent stability and accuracy problems in SEM analyses, as they usually require a considerable sample size, even up to 200 to guarantee stable parameters (Nachtigall et al., 2003); however, bootstrapping techniques included in PROCESS overcome this issue (Hayes, 2018); (b) our categorical independent variable that increases the complexity of SEM analyses but are ‘truly' modeled as dummy variables in regression analyses with PROCESS (Nunkoo and Ramkissoon, 2011); and (c) studies proved that PROCESS and SEM that generally produce same results, with the latter requiring complex programming that might lead to errors (Hayes et al., 2017; Hayes and Rockwood, 2020).

##### 2.4.2.2. Cluster analyses

To explore the different profiles in this NPO context, we conducted a cluster analysis. This data mining technique allows to identify and classify groups (clusters) based on information found within the data, describing samples and their relationships (Larose, 2005). Participants belonging to the same cluster show a similar pattern while being as dissimilar as possible from participants who integrate the other clusters. We analyzed which profiles can be detected based on participants' perception of intrapersonal conflict (role conflict, role ambiguity, and role overload), conflict consequences (job satisfaction, job performance), and sociodemographic variables (age, seniority in the NPO, seniority in the project).

To contrast our hypotheses, we conducted two different cluster algorithms, hierarchical cluster, to determine the optimal number of clusters in the data, and k-means cluster, which establishes the presence of clusters by finding their centroid points. A centroid point is the average of all the data points in the cluster. By iteratively assessing the Euclidean distance between each point in the dataset, each one can be assigned to a cluster. The centroid points are random to begin with and will change each time as the process is carried out. Both cluster analyses were conducted using Z scores as measurement scales differed in some variables (Mohamad and Usman, 2013).

Finally, a between-groups one-way ANOVA was conducted to determine whether there are significant differences on the dependent variables across the different clusters.

## 3. Results

Descriptive statistics including means and standard deviations for primary study variables appear in Table 1. Before testing the SMM model, including those for paid staff and volunteers separately. Correlations were computed within a bivariate framework and are also displayed in Table 1. Results indicate that, in both groups, intrapersonal conflicts' correlations (role conflict, role ambiguity, and role overload) are significant and positive. Performance and job satisfaction also correlate significantly and positively. However, intrapersonal conflicts' correlations with performance and job satisfactions are significant and negative.

### 3.1. Serial mediation model

**Figure 2:**
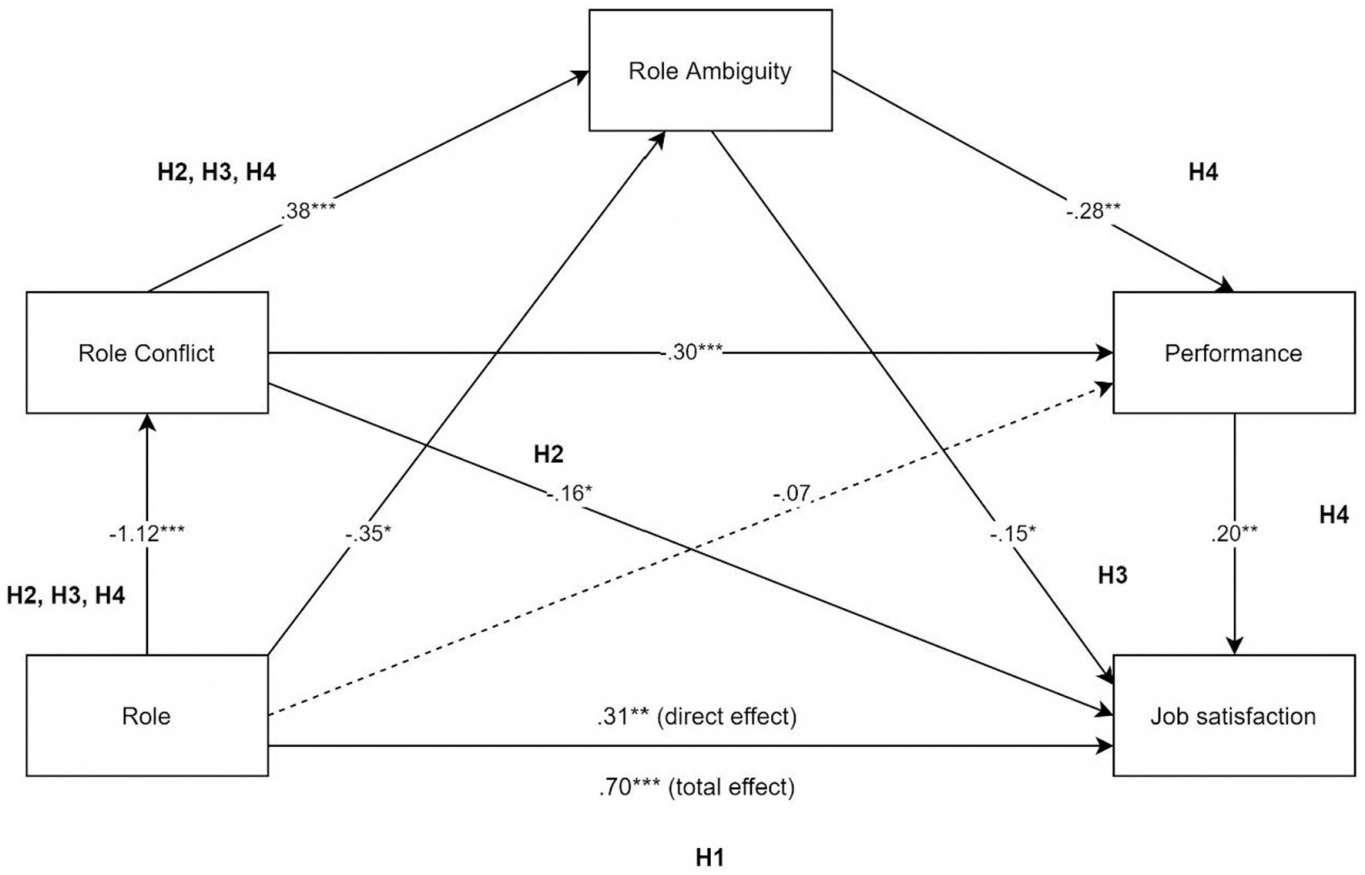
Complete serial multiple mediation model and path coefficients.^1^^***^p < 0.001; ^**^p < 0.01 ^*^p < 0.05, *B* values are presented in the model.^2^ ^1^This model was designed based on López-Cabrera et al. (2020). Additional models including different and same variables were also tested, obtaining non-significant results. ^2^This model presents regression coefficients (B), so values can be larger than 1. In this case, as the independent variable is categorical, path a (role-role conflict) reflects the difference between volunteers and paid staff on role conflict. This coefficient is negative due to the order of variables; PROCESS is comparing volunteers vs. paid staff, and the latter reports more role conflict, as it is presented in Table 1.

All path coefficients were calculated using regression analysis with PROCESS for SPSS (Hayes, 2018). This approach has advantages compared with other statistical methods, as it enables the isolation of each mediator's indirect effect as well as the complete indirect effect of the serial mediators (Van Jaarsveld et al., 2010). The complete serial mediation model is presented in Figure 2, and regression coefficients, standard errors, and model summary information for the serial multiple mediator model are presented in Table 2.

**Table 2:** Regression coefficients, standard errors, and model summary information for the serial multiple mediator model.

**Antecedent**	***M1*** **(RC)**			***M2*** **(RA)**			***M3*** **(PERF)**			***Y*** **(JSAT)**		
	***B***	***SE***	***p***	***B***	***SE***	***p***	***B***	***SE***	***p***	***B***	***SE***	***p***
*X* (R)	−1.12	0.15	0.000	−0.35	0.17	< 0.05	−0.07	0.18	>0.05	0.31	0.13	< 0.05
*M1* (RC)	-	-	-	0.39	0.09	0.000	−0.30	0.10	< 0.05	−0.16	0.07	< 0.05
*M2* (RA)	-	-	-	-	-	-	−0.28	0.10	< 0.01	−0.15	0.07	< 0.05
*M3*(PERF)	-	-	-	-	-	-	-	-	-	0.20	0.07	< 0.01
	*R*^2 =^ 0.32 *F* (1,111) =53,24; *p =* 0.000			*R*^2 =^ 0.32 *F* (2,110) =25.57; *p =* 0.000			*R*^2 =^ 0.31 *F* (3,109) =16.42; *p =* 0.000			*R*^2 =^ 0.48 *F* (4,108) =24,49; *p =* 0.000		

R, role; RC, role conflict; RA, role ambiguity; PERF, performance; JSAT, job satisfaction; M, mediator.

Indirect effects are tested by means of a bootstrapping procedure (10,000 subsamples), addressing some weaknesses associated with the Sobel test (Van Jaarsveld et al., 2010). Results for indirect effects along with the 95% bias-corrected bootstrapped confidence intervals for the path estimates are presented in Table 3. The results demonstrate that the 95% confidence intervals for all hypothesized indirect effects do not contain zero, confirming the proposed constructs (role conflict, role ambiguity, performance) as serial mediators between role and job satisfaction (Terglav et al., 2016).

**Table 3:** Significant indirect effects of the serial multiple mediation model.

**Indirect path**	**Effect**	***SE***	**95% CI^a^**
Total	0.4	0.1	[0.21, 0.59]^*^
R → RC → JSAT	0.18	0.08	[0.03, 0.36]^*^
R → RA → JSAT	0.05	0.04	[−0.003, 0.16]
R → PERF → JSAT	−0.01	0.03	[−0.09, 0.05]
R → RC → RA → JSAT	0.06	0.04	[0.003, 0.16]^*^
R → RC → PERF → JSAT	0.06	0.03	[0.01, 0.14]^*^
R → RA → PERF → JSAT	0.02	0.02	[−0.001, 0.06]
R → RC → RA → PERF → JSAT	0.02	0.01	[0.003, 0.06]^*^

a 10,000 bootstrap samples for bias-corrected bootstrap confidence intervals. R, role; RC, role conflict; RA, role ambiguity; PERF, performance; JSAT, job satisfaction.

* Significant indirect effect, as CI intervals do not contain value 0.

To test H1, we analyzed the total effect of role (paid staff and volunteers) on job satisfaction (*b* = 0.70, *SE* = 0.11, *p* = 0.000), which is significant. This indicates there is a difference in perceived job satisfaction between paid staff (*M* = 3.60; *SD*=0.61) and volunteers (*M* = 4.30; *SD*=0.52). As hypothesized, paid staff report less job satisfaction. The indirect effect is also significant (*b* = 0.18, *SE* = 0.08, *CI*= [+0.03; +0.36]). Thus, H1 is accepted.

According to the results, there is a difference in perceived role conflict between paid staff (*M* = 2.87; *SD*=0.84) and volunteers (*M* = 1.75; *SD*=0.73). Role conflict mediates the relationship between role and job satisfaction; as role conflict increases, job satisfaction decreases. Results demonstrate that role predicted role conflict (*b* = –1.11, *SE* = 0.15, *p* = 0.000) and that role conflict predicted job satisfaction (*b* = −0.16, *SE* = 0.07, *p* < 0.05). The significance test required prediction of an indirect effect of role influencing job satisfaction. Results show a significant indirect effect (*b* = 0.18, *SE* = 0.08, *CI* bootstrapped 95% *CI*= [+0.03; +0.36]), supporting H2.

Also, results demonstrate that role conflict and role ambiguity, together, mediate the relationship between role and job satisfaction. The perception of role conflict increases role ambiguity among participants, which decreases job satisfaction. Role predicted role conflict and role conflict predicted role ambiguity (*b* = 0.38, *SE* = 0.09, *p* = 0.000). Finally, role ambiguity predicted job satisfaction (*b* = –0.15, *SE* = 0.07, *p* < 0.05). As indirect effect analysis was also significant (*b* = 0.06, *SE* = 0.04, bootstrapped 95% *CI*= [+0.003; +0.16]), H3 is supported.

Finally, to test our H4, the complete serial multiple mediation model was tested. H4 states that role (paid staff, volunteer) affects the reported job satisfaction, while this relationship is mediated by role conflict, role ambiguity, and job performance. The indirect effect of role on job satisfaction through the mediation of role conflict, role ambiguity, and performance was significant (*b* = 0.02, *SE* = 0.01, *CI*= [+0.003; +0.06]). Therefore, the results of the analysis show that there is a difference in perceived role conflict between paid staff and volunteers, as previously mentioned. Role conflict relates to higher role ambiguity, which relates to lower higher levels of performance. At the same time, performance is associated with higher levels of job satisfaction. Thus, H4 is supported; paid staff and volunteers' differences in job satisfaction are serially mediated by role conflict and role ambiguity and performance. This serial mediation is partial as direct effect of role on job satisfaction is still significant, as stated in H1. However, direct effect (*b* = 0.31, *SE* = 0.12, *p* < 0.01) is smaller than total effect (*b* = 0.70, *SE* = 0.11, *p* < 0.001). As a result, the study accepts all its hypotheses.

Additionally, this serial multiple mediation model emerged with additional associations. Only three indirect effects were not significant: (a) role, role ambiguity, job satisfaction; (b) role, performance, job satisfaction; and (c) role, role ambiguity, performance, satisfaction. Complete results are presented in Table 3.

As can be observed, the non-significant indirect effects do not include role conflict as a moderator. These results suggest that the perception of having to fulfill the duties of two contradictory positions or the perceived incompatibility between expectation and reality from a job or position are a key mediator between roles and role ambiguity (lack of clarity of one's job profile), performance, and job satisfaction.

### 3.2. Cluster analyses

Considering our second aim, cluster analyses were conducted to explore the existence of homogeneous profiles among members of NPOs, inferring patterns based on their perceptions of the organizational context and personal characteristics. Particularly, we analyzed which profiles report significantly higher levels of job satisfaction and what factors are related to these perceptions.

First, we conducted hierarchical cluster analysis using Euclidean distance as an interval measure and Ward's method, using the F value (like in ANOVA) to maximize the significance of differences between clusters. Based on the dendrogram and agglomeration schedule, a two-, three-, and six-cluster solution were found. These solutions were explored using k-means clusters and exploring graphically the cases dispersion graphics using a factorial analysis, based on principal components analysis as extraction method and Varimax with Kaiser normalization as rotation method (Factor 1—-*Perception of the organization*: role conflict, role ambiguity, satisfaction, role overload, and performance; Factor 2—*Internalization of organizational structure and procedures*: age, seniority in NPO, seniority in current project).

**Figure 3:**
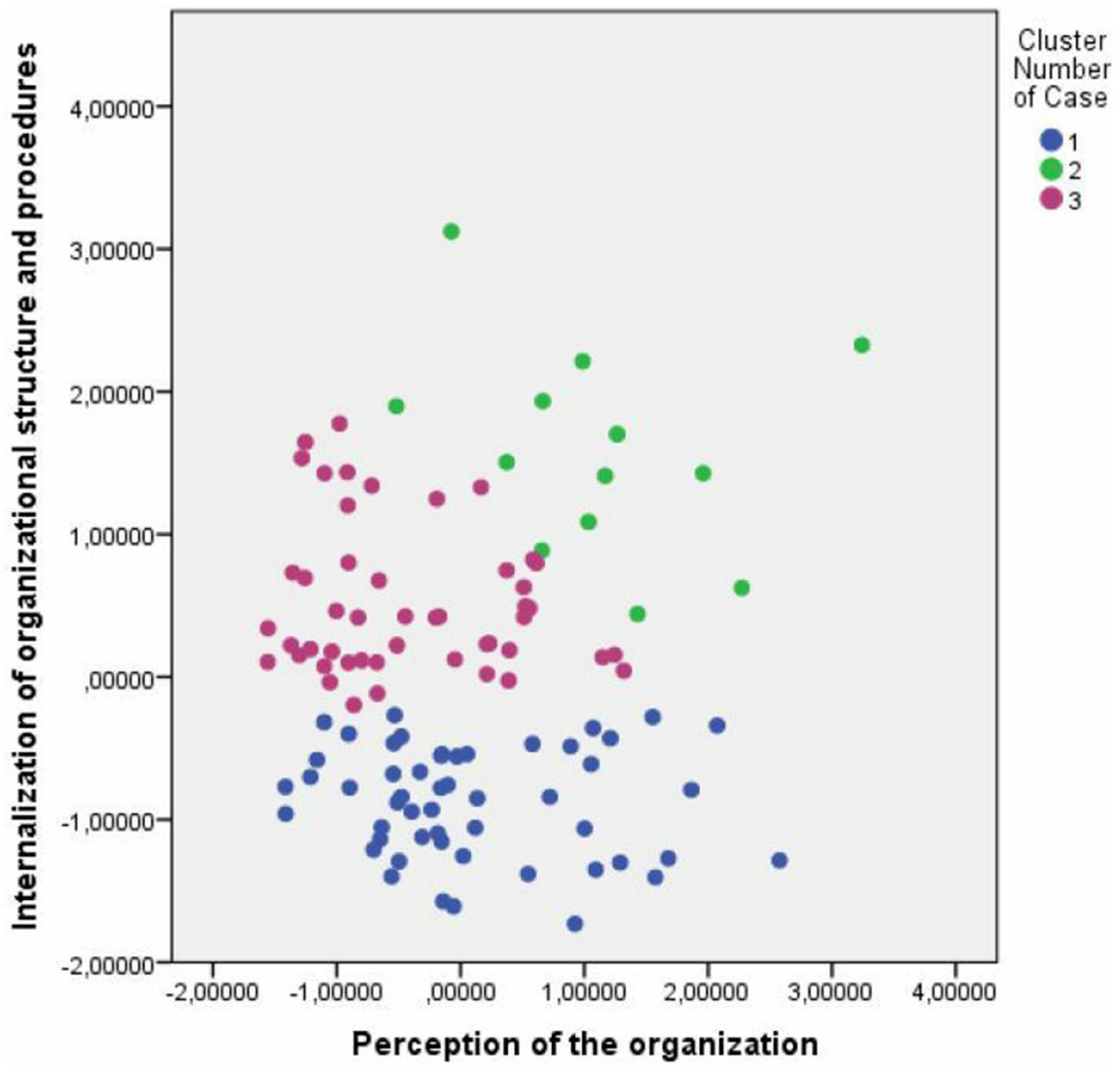
Three-Cluster solution.

**Figure 4:**
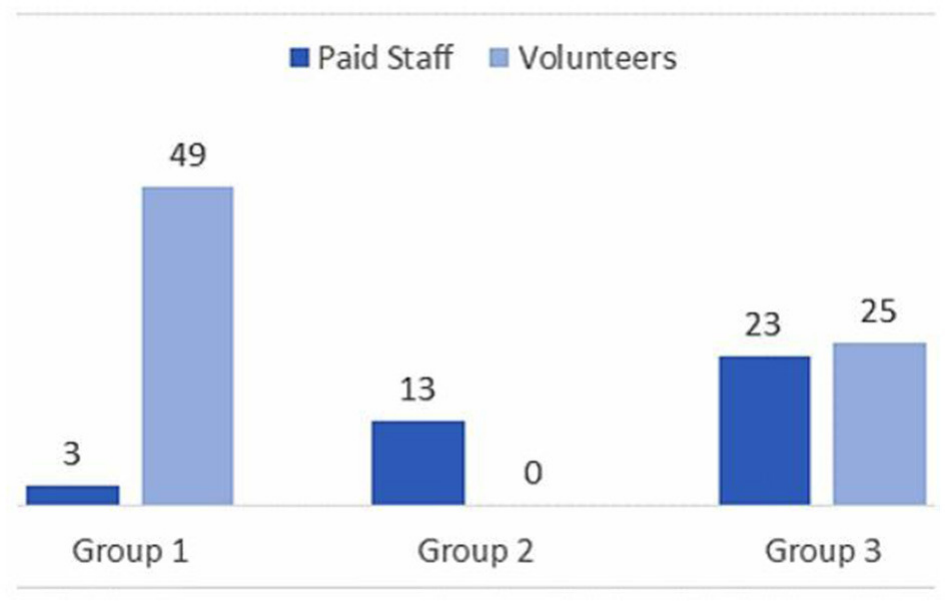
Paid staff and volunteer's distribution among profiles.

**Figure 5:**
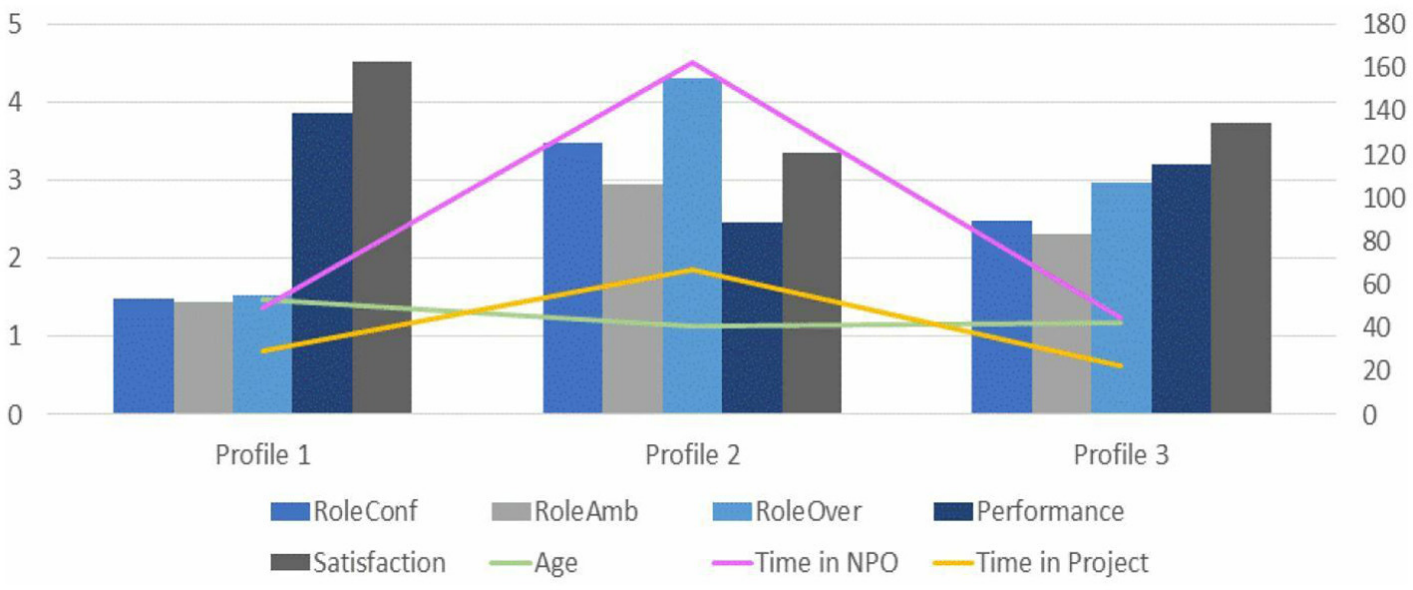
Profiles based on the three-cluster solution. Time in NPO and project was measured in months. Age was measured in years. Variables are presented in order, from left-right, up-down.

The three-cluster solution improved on the two-cluster model, by splitting a cluster into two distinctive groups. The six-cluster solution, however, did not create any organizationally additional relevant segments, as mostly included a very limited number of cases. Therefore, the three-cluster solution was selected (see Figure 3 for three-cluster dispersion graphics and NPOs' members' distribution among them). Graphs comparing the paid staff volunteers distribution among the three clusters solution are presented in Figure 4. Figure 5 shows the profiles based on the three-cluster solution.

Finally, a between-groups one-way ANOVA was conducted to determine whether there are significant differences in the dependent variables across the different clusters (Table 4). Multiple comparisons between the mean scores of the different clusters (or profiles) are reported based on *post-hoc* Tukey HSD as well as the effect size, calculated based on eta squared.

**Table 4:** Means, standard deviations, and one-way ANOVA in perception of the organization and internalization of organizational structures and procedures.

**Measure**	**Profile 1**		**Profile 2**		**Profile 3**		***F* (2,110)**	**η^2^**	**Multiple comparisons (*Post-hoc* Tukey HSD)**
	***M***	***SD***	***M***	***SD***	***M***	***SD***			
Role conflict	1.49	0.51	3.49	0.74	2.47	0.76	59.35^***^	0.52	1-2^***^; 1-3^***^; 2-3^***^
Role ambiguity	1.44	0.56	2.95	0.87	2.32	0.69	37.62^***^	0.40	1-2^***^; 1-3^***^; 2-3^**^
Role overload	1.53	0.59	4.30	0.63	2.97	1.03	77.12^***^	0.58	1-2^***^; 1-3^***^; 2-3^***^
Performance	3.87	0.56	2.46	0.61	3.20	0.70	31.05^***^	0.36	1-2^***^; 1-3^***^; 2-3^***^
Job satisfaction	4.53	0.38	3.35	0.57	3.74	0.52	51.99^***^	0.49	1-2^***^; 1-3^***^; 2-3^*^
Age	52.81	16.03	40.46	6.00	42.25	13.16	8.61^***^	0.13	1-2^*^; 1-3^***^; 2-3
Time in NPO	49.52	55.24	162.23	57.30	44.27	54.10	25.38^***^	0.32	1-2^***^; 1-3; 2-3^***^
Time in project	29.60	28.22	66.77	46.43	22.31	19.54	13.26^***^	0.19	1-2^***^; 1-3; 2-3^***^

*** p < 0.001;

** p < 0.01

* p < 0.05.

Profile 1 is composed mostly by volunteers, 49.23%, and only 5.77% of paid staff. On the contrary, in Profile 2 members are 100% paid staff. Profile 3 is the most balanced group, regarding the distribution of organizational roles. It is composed of 47.92% of paid staff and 52.08% of volunteers. Results demonstrate that there are significant differences in all the variables measured among the three profiles established by the previous cluster analyses. To analyze whether there are significant differences between each group, multiple comparisons analyses were conducted. Results are summarized in Table 4.

Mean comparisons on *role conflict, role ambiguity*, and *role overload* demonstrate that participants clustering in Profile 2, all of them paid staff, report the highest mean scores compared with Profile 1 and Profile 3. Indeed, Profile 1, composed mostly by volunteers, reports the lowest mean scores for these variables. Means comparisons among the three profiles are statistically significant. Regarding *job satisfaction* and *performance*, results demonstrate that there are significant differences among the three profiles. Profile 1 presents the highest mean scores for both variables; however, participants clustered in Profile 2 present the lowest score for job satisfaction and perception of performance.

Finally, results show significant differences on *age, time in the NPO and in projects*. Regarding age, Profile 1, in which volunteers clearly predominate, presents the highest mean scores. Indeed, there are significant mean scores between Profiles 1 and 2, and Profiles 1 and 3, respectively. Indeed, age seems to be a main difference between Profiles 1 and 3 in which there are majority of volunteers. Profiles 2 and 3 do not present significant differences on age. Regarding seniority in NPOs and in actual projects, Profile 2 presents the highest scores. This is coherent with the organizational characteristics, as paid staff have stable job positions (Profile 2), compared to volunteer's flexible participation in NPO's activities (Profiles 1 and 3), who present a lower mean score for time in NPO and projects. These differences are significant when comparing Profiles 1 and 2 and Profiles 2 and 3, respectively. Table 5 summarizes the three profiles identified in NPO.

**Table 5:** Profiles in NPO: a summary.

**Profile 1** Senior volunteers	More than 4 years collaborating in the NPO and less than 2 years working in their project. They report very high levels of job satisfaction and perception of performance. Role conflict, role ambiguity, and role overload reported levels are quite low: - Based on their perceptions, they do not have to deal with duties of contradictory positions (role conflict). - They consider they have received clear information about their tasks and their methods, or at least it is not considered a problem (role ambiguity). - They do not consider they have problems dealing with all their work (role overload).
**Profile 2** Long-term paid staff	More than 13 years working in the NPO, more than 5 years working on their current project. They report medium levels of job satisfaction and medium–low performance. Role conflict, role ambiguity, and role overload reported levels are quite high: - Based on their perceptions, they consider that they have dealt with duties of contradictory positions (role conflict). - They consider they do not have clear information about their tasks and their methods, or at least, which affects their work (role ambiguity). - They also report very high levels of role overload, they do not have enough resources (for example, time) to deal with all their work (role overload). - Probably, they are the most affected by professionalization-related organizational changes.
**Profile 3** Volunteers and recent paid staff	Less than 4 years working/collaborating in the NPO, < 2 years working in their current projects. They report medium-high levels of job satisfaction and performance. Role conflict, role ambiguity, and role overload reported levels are quite low but higher than in profile 1. - Based on their perceptions, they do not have to deal with duties of contradictory positions (role conflict). - They consider they have received clear information about their tasks and their methods, or at least it is not considered a problem (role ambiguity). - They do not consider they have problems dealing with all their work (role overload).

## 4. Discussion

NPOs are a complex working context whose main characteristic resides in the dichotomy between paid staff and volunteers (Netting et al., 2005). Despite the benefits that this organizational feature has for goal achievement, it also entails difficulties not only for interaction between both groups, leading to interpersonal conflicts (Kreutzer and Jäger, 2011; McAllum, 2018; López-Cabrera et al., 2020), but also to guarantee a complete understanding of their own role (King, 2017). Also, it can be a challenge to HR and volunteers' managers, as both groups usually have different perspectives of their working context and different needs, for example, to achieve job satisfaction (López-Cabrera et al., 2020). Indeed, managing diversity is a main challenge not only for Organizational Psychology, but also for organizations and international institutions, which has turned out to be even more crucial during the last years, considering the relevance of NPO's work in times of need.

The aim of this study was to understand the existent differences between paid staff and volunteers, regarding their perceptions of the organizational context, and personal characteristics. Particularly, we explored (1) which factors may contribute to promote job satisfaction among NPOs members and (2) whether considering these factors, it is possible to create an additional categorization of NPOs workforce that permits HR and volunteering managers to anticipate problems and promote job satisfaction.

Results demonstrate that paid staff and volunteers report significantly different job satisfaction, indeed, paid staff report less job satisfaction. These results are consistent with previous research supporting this difference (Rimes et al., 2017). However, these differences are traditionally explained based on the importance that particularly volunteers give to values, which in most NPOs is related to helping others (Bang et al., 2013). The present study goes a step further contributing to the understanding of how and why these differences exist among both groups, analyzing the effect of role conflict, role ambiguity, and performance that prove to serially mediate the effect of role on job satisfaction.

Paid staff reported higher levels of role conflict and role ambiguity, as they considered they must fulfill the duties of two different positions which they considered unclearly defined. Considering this serial mediation effect, our results are quite relevant particularly for paid staff, who have been usually overlooked when analyzing NPOs even though they are, indeed, less satisfied with their job than volunteers.

Boundaries between paid staff and volunteers' duties are, in real practice, difficult to differentiate, particularly during the last years (McAllum, 2018). This organizational context is changing due to the professionalization process that NPOs are facing to cope with economic challenges (Müller-Stewens et al., 2019; Clausen, 2021). Indeed, this uncertainty entails problems not only for interaction between both groups but also to guarantee a complete understanding of their own role. Consequently, both groups have reported role conflict and role ambiguity as main concerns for their daily development of their work (López-Cabrera et al., 2020). Therefore, paid staff and volunteers are not homogenous roles, indeed, their even more complex than expected. This results in discrepancies not only between groups but also at the person-role level.

Regarding performance, previous studies pointed out that especially role conflicts have a negative effect on performance (Amilin, 2017). However, the results of the serial mediation model presented in this study indicate that performance has a buffering effect of the negative influence of role conflict and role ambiguity on job satisfaction. This can be explained by the fact that in NPOs, a positive perception of project performance implies that their social interventions are considered effective. Results demonstrating that volunteering job satisfaction is higher are consistent, based on the relevance that values have particularly for volunteers. However, in this study, this result demonstrates that, to some extent, the social relevance of NPOs activity is a protective factor to the overall workforce, including paid staff.

This study also analyzes the existence of homogenous patterns or profiles that may help HRM to provide specific support and foresee problems, being able to act as soon as possible, besides the formal classification in NPOs paid staff and volunteers. According to cluster analyses, Profiles 1 and 2 reflect quite consistently different perceptions from volunteers and long-term paid staff, respectively. Indeed, Profile 1 reports higher scores for those variables related to positive perceptions of the NPO (job satisfaction, performance). On the contrary, Profile 2 participants report higher scores for those measures related to conflict (role conflict, role overload, role ambiguity). These differences are consistent with both our first research question results and with previous studies (López-Cabrera et al., 2020).

However, Profile 3 results may be very relevant for HRM management purposes. Results indicate that this group, composed of volunteers and paid staff, may be a “transition” group; it comprises volunteers and, based on time in the organization, recent paid staff. Overall, this group presents medium–low scores in most variables, which can be an opportunity to solve problems on early stages as well as promote job satisfaction. Therefore, paying attention to Profile 3 can also provide a perspective on how roles evolved along time in this NPO. Indeed, time in NPOs seems to be determinant to significantly increase stable paid staff results related to role conflict, role ambiguity, and role overload compared with volunteers. This increase can be explained based on social comparison theory (Festinger, 1954) and equity theory (Adams, 1963, 1965). Paid staff create their role perceptions and evaluate their contributions, rewards, and efforts to some extent comparing them with volunteers' roles. If, despite their seniority in the organization, paid staff still perceive discrepancies regarding their expectations and their real tasks and activities, with the organization doing nothing to clarify them, there is no perception of fairness because of that social comparison. This unbalance can lead to both intrapersonal conflicts but also interpersonal conflicts, as discussed by López-Cabrera et al. (2020). On the contrary, volunteers report higher scores for job satisfaction and performance, which are positive measures for both the organization and volunteers. It is also remarkable the differences in age between Profiles 3 and 1. Volunteers in Profile 1 are senior volunteers, which implies that they may have more work experience in their job positions. Therefore, they can also manage better possible role conflict, role ambiguity, and role overload, and consequently, they report better job satisfaction.

Overall, it is crucial that HR management understands how paid staff and volunteers differently perceived their organizational context and the impact that those perceptions have on their job satisfaction; therefore, they will be able to prevent intrapersonal conflicts and promote policies that fit their employees (both paid staff and volunteers) needs. However, if they only focus on the fixed paid staff–volunteers categorization, it would be difficult to manage the heterogeneity that these groups present. Focusing only on these “formal labels” overlooks crucial information to guarantee, on the one hand, employees' full understanding of their daily duties and, on the other hand, the efficiency of managers and supervisors in their monitoring activities.

### 4.1. Theoretical and practical implications

This research has important practical and theoretical implications, particularly on HR management and NPOs understanding. First, it contributes to understand the mechanism that promotes differences on job satisfaction among paid staff and volunteers based on role conflict, role ambiguity, and performance. In this regard, role conflict and role ambiguity have a negative impact on job satisfaction, particularly for paid staff who report higher levels of intrapersonal conflict. On the contrary, performance buffers this negative effect. This information is quite relevant to create HR policies that decrease volunteers and, particularly, paid staff's uncertainty regarding their roles. A detailed description of their positions and the tasks related to them could be a way to promote job satisfaction in NPOs workforce, also by increasing their perception of good performance. This is particularly relevant considering the organizational changes that NPOs are facing due to their professionalization process, which increases uncertainty among paid staff and volunteers.

Also, this study provides relevant practical information on different profiles in NPOs. These results reinforced the differences between paid staff, who report more intrapersonal conflict and less performance and job satisfaction, and volunteers, who reported the opposite tendency. Results demonstrate the existence of an intermediate group, composed of recent paid staff and volunteers. Considering this information, HR manager can design strategic initiatives oriented to attend to each profiles' needs, such as courses or supporting procedure.

Ultimately, these results can contribute to improve Decision Making and Management Practices in NPOs, improving internal processes and meeting the specific needs of accountability and legitimacy for stakeholders. Considering the Profiles obtained in our analysis, anticipating problems regarding Profile 2 (long-term paid staff) during decision-making processes is possible. Profile 2 seems to be the group that faces more intrapersonal conflicts may be more affected by organizational changes. Therefore, decision-making processes should be oriented to decrease their workload and uncertainty on understanding which are their duties. As they are usually the most experienced members of each project, they must cope with stress not only derived from their own position, but also from coaching and supervising new employees. Therefore, measures oriented to make it possible to find new challenges, such as collaborating with other projects, can help to reduce intrapersonal conflicts and improve job satisfaction and performance, as they must focus on different and perhaps more motivating demands. Results also contribute to possible decision-making processes regarding Profile 3 (volunteers and recent paid staff). In this case, prevention is crucial, as this group includes members of the organization that report medium–high levels of job satisfaction and performance but role conflict, role ambiguity, and role overload reported levels are quite low. In this case, decision making should focus on providing information and appropriate job analyses in order to state from the beginning the task and duties associated with each role. Also, policies that contribute to keep improving job satisfaction and performance to high levels are also important, promoting that these volunteers join Profile 1 and new paid staff change the dynamics of Profile 2 when they become senior staff reducing intrapersonal conflicts and improving job satisfaction and performance.

### 4.2. Potential limitations and future studies

This study has some limitations that, although can be considered as opportunities for future research, must be also considered to generalize the results obtained.

First, the cross-sectional design of this research restricts the extent that cause–effect relationship can be assumed from the results. Although this limitation should be considered, it still provides valuable information for HR practices that is coherent with previous research on the matter, including qualitative studies. Future studies should consider setting out longitudinal studies being able to analyze changes among profiles in NPOs. Second, this research is based on self-reported measures, as we intend to analyze paid staff and volunteers' perceptions of their working context. Therefore, common method bias can be a concern in this study. However, some measures were taken to minimize this bias, including the use of established scales, different sets of instructions, and filler items for each variable and scales were organized avoiding the replication of the order of the hypothesis (Podsakoff et al., 2012; Alfes et al., 2015; Terglav et al., 2016). Future studies should also consider a multi-source and multilevel design, including, for example, users and HR data as performance measures and considering different projects as working groups within a common organization.

Third, regarding the sampling method, our study was conducted in an only NPO branch, using a convenience sample. Although participants were invited and they freely decided to participate, which added some randomization, it is not possible to guarantee that the sample is fully representative of NPOs workforce. However, as previously mentioned, results are consistent with previous research in the third sector. Also, as a positive remarkable aspect of our participant NPO, it is a worldwide organization that replies to the same functional and hierarchical structure in every national and international location. Therefore, results can potentially be generalized to all these divisions around the world. Future studies should replicate the analysis in different NPOs with different cultures, using a random sampling method, to contribute to generalize our conclusions to different organizations.

## 5. Conclusion

This study contributes to the understanding of the complex NPOs workforce, focusing on the perceived differences between two different roles: paid staff and volunteers. NPOs represent a great example of how challenging diversity management can be in organizations, particularly considering its different sources, that include not only personal characteristics but also the organizational structure itself or emergent profiles of organizational members.

First, from a theoretical perspective, this study improves the comprehension of the mechanisms and relations that lead to differences between paid staff and volunteers regarding job satisfaction, performance, role conflict, and role ambiguity. Results demonstrate that paid staff and volunteers report significantly different job satisfaction; indeed, paid staff report less job satisfaction. Paid staff report more intrapersonal conflicts and less performance and satisfaction, whereas volunteers' results are completely opposed, reporting higher levels of perceived performance and job satisfaction and less intrapersonal conflicts. Role conflict relates to higher role ambiguity, which relates to lower higher levels of performance. At the same time, performance is associated with higher levels of job satisfaction.

Results also demonstrate that paid staff and volunteers' differences on job satisfaction are serially mediated by role conflict and role ambiguity and performance. Second, as main practical implication, results demonstrate the existence of an additional profiles distribution that reflects more adequately the reality of NPOs, besides the formal roles that coexist in these organizations (paid staff and volunteers). These profiles can constitute a crucial analytic tool to HRM; in this context as managing this information, HR managers can design policies and initiatives according to the real needs of organizational members, optimizing paid-staff and volunteer's management to increase their satisfaction and even reduce NPO dropout.

Nowadays, NPOs have demonstrated to develop a crucial supporting labor for society particularly during difficult times such as COVID-19. However, promoting its understanding is a challenge for Organizational Psychology. This study highlights the complexity of NPOs not only regarding their structure, interpersonal relationships among members, or at person-role level but also within roles. Notwithstanding, these results may facilitate both the understanding and management of this intricate, but fundamental to fulfill the important purposes of NPOs' workforce.

## Data availability statement

The raw data supporting the conclusions of this article will be made available by the authors, without undue reservation.

## Ethics statement

The studies involving human participants were reviewed and approved by the Ethics Committee of the Faculty of Psychology (University of Seville, Spain) and the Ethics Committee of the participant non-profit organization. The patients/participants provided their written informed consent to participate in this study.

## Author contributions

All authors contributed to the conception and design of the work and the acquisition, analysis, and interpretation of data. They drafted the work and revised it critically. The authors gave the final approval of the manuscript before the submission and participated at every stage of the research process.
